# The beef terroir: moving from a commodity and its role in human evolution to a new paradigm of health, epigenetics, and sustainability: a review

**DOI:** 10.3389/fnut.2026.1839131

**Published:** 2026-06-12

**Authors:** Rodrigo Arias, Alejandro Velásquez

**Affiliations:** 1 Faculty of Agricultural and Food Sciences, Institute of Animal Production, Universidad Austral de Chile, Valdivia, Chile; 2 Volcanic Soils Center, Universidad Austral de Chile, Valdivia, Chile; 3 Department of Agricultural Sciences and Aquaculture, Faculty of Natural Resources, Universidad Católica de Temuco, Temuco, Chile; 4 Research Nucleus in Food Production, Universidad Católica de Temuco, Temuco, Chile

**Keywords:** beef, bioactive compounds, epigenetics, grazing management and animal production, regenerative grazing

## Abstract

Red meat intake has been associated with the evolutionary journey of human beings, shaping metabolic pathways, cognitive development, and social complexity. Beyond its role as a nutrient source, meat, beef particularly, represents a biologically complex matrix whose composition reflects the ecological and production contexts in which the animal was raised. This review integrates evidence from evolutionary biology, nutritional science, epigenetics, and metabolomics to reframe the role of beef within human development and modern food production systems. We propose the concept of “beef terroir” to describe how soil–plant–animal interactions influence animal performance and beef quality, and, consequently, human health. Differences between pastoral and industrial (feedlot) production systems are critically examined alongside alternatives such as plant-based systems. Finally, we identify priority research areas required to align livestock production with human health and sustainability goals.

## Evolutionary significance of animal-source foods intake in human beings

There is consensus that the consumption of meat, fat and, marrow (MFM), became a routine part of the hominin diet at least 2.6 million years ago. The evidence collected shows the coincidence with the development of stone tools and the tripling of brain size over the next 3 million years (1–3). These foods provided the concentrated energy, high-quality (available) protein, and critical micronutrients, particularly vitamin B12, zinc, iron, and long-chain fatty acids like EPA and DHA, needed to sustain the large human brain’s function. Nevertheless, today it is believed that not only meat but also fat and marrow intake have been crucial for human evolution and have gained greater attention. For example, archeological sites (from 2.5–2.0 million years ago) show systematic evidence of marrow extraction, with characteristic spiral fractures and percussion marks on bones indicating intentional processing (4). Early hominins developed specialized stone tools specifically for accessing marrow, demonstrating the high value placed on these nutrient-dense tissues. Fat represents the most energy-dense macronutrient available to early humans. While lean beef provides 100–200 kcal/100 g, beef fat contains approximately 900 kcal/100 g, that is, about triple the energy density of protein or carbohydrates (4). This energy density was particularly crucial for supporting the high metabolic demands of enlarged brains during human evolution. On the other hand, analysis of bone assemblages from archeological sites such as Olduvai Gorge suggests that early hominins prioritized marrow extraction over muscle meat acquisition, indicating that fat maximization, rather than protein, was often the primary criterion for carcass processing decisions (4).

The inclusion of MFM in the diet matches with the “expensive tissue hypothesis,” which states that the human brain consumes ~20% of resting metabolic energy. Thus, early humans privileged a nutrient-dense diet (high-calorie), such as that provided by MFM, which cannot be easily supplied by plant foods alone, especially in a pre-agricultural era. In addition, it has been demonstrated that relative brain size correlates positively with dietary quality (6, 7). Consequently, the shift toward energy-dense foods such as MFM reduced gut size while supporting brain expansion (8). In addition, human metabolism exhibits remarkable adaptations for fat utilization, including enhanced ketogenesis and fatty acid oxidation, which align with the social behavior of hunting, in which a high rate of failure could lead to extended periods of fasting. These adaptations allowed early humans to maintain cognitive function during periods of food scarcity by utilizing ketone bodies as alternative brain fuel (8). The omega-3 fatty acids (EPA/DHA) were particularly important for supporting the rapid brain expansion observed in the genus Homo. Some researchers have argued that without routine access to MFM, it is highly unlikely that early humans could have achieved their unusually large and complex brains while simultaneously continuing their evolutionary trajectory as large, active, and highly social primates (9, 10). The shift to MFM also drove social evolution: cooperative hunting of large game fostered altruistic and social skills (2).

The evolutionary adaptations discussed herein were beneficial in ancestral environments. However, the modern human diet and lifestyle have changed enormously since then, especially after cattle domestication, and the postindustrial era, and the consumption of a high proportion of processed and ultra-processed foods, including meats, carbohydrates, and omega-6-rich vegetable oils, among sedentary populations may contribute to actual metabolic disorders observed in modern society (11). For some researchers, the key distinction lies between the intermittent, unprocessed meat consumption of our ancestors and the continuous, highly processed meat intake common in industrialized societies (8).

## Nutritional profile of beef and health risks

Beef is a nutrient-dense matrix that is difficult to replicate from plant sources alone as a whole. It provides all proteinogenic amino acids in balanced ratios, along with taurine, *β*-alanine, creatine, carnosine, and 4-hydroxyproline, among other bioactive compounds that play crucial roles in antioxidative reactions, neurological function, muscular performance, and cardiovascular health in humans (12). These bioactive compounds are either absent from or present in negligible amounts in plant foods (12, 13). For example, 30 g of dried beef can provide 80.4 mg taurine (meeting 107% of daily requirements), 608 mg carnosine (100% of daily requirements), and substantial amounts of creatine (12). Beef is also a leading source of highly bioavailable heme Fe, Zn, Se, and vitamin B12 (14). A study analyzing adults (aged 60+) found that beef consumers had significantly higher intakes and greater adequacy of protein, Zn, Ca, Fe, vitamin B12, and several other micronutrients than non-consumers (15). Dietary modeling studies suggest that the lowest-cost nutrient-adequate diets in the U. S. A. include MFM such as beef liver (16). For children in low- and middle-income countries, MFM are particularly important because they contribute to cognitive development, and studies show compelling, though not yet conclusive, benefits of meat consumption in reducing stunting and cognitive impairment (17, 18).

For more than 50 years, red meat and processed red meat have been associated with increased risks of all-cause mortality, cardiovascular disease, and metabolic disease. However, there are practically no cause-and-effect studies supporting those (19–21). Nowadays, there is a shift in the paradigm highlighting the importance of the MFM in human health. In this context, it has been proposed that an additional 100 g/day of red meat was associated with a 17% higher risk of type 2 diabetes, a 15% higher risk of coronary heart disease, a 14% higher risk of hypertension, and a 12% higher risk of stroke (22). For processed meat specifically (per 50 g/day increase), the risks were even steeper: RR 1.37 for T2DM, 1.27 for Coronary Heart Disease (CHD), and 1.15 for all-cause mortality (22). However, the strength of this evidence has been largely debated. A prominent 2019 series in the *Annals of Internal Medicine* (NutriRECS) systematically reviewed both observational studies and randomized controlled trials and concluded that the evidence linking red meat to cardiometabolic and cancer outcomes was of low to very low certainty, noting that effect sizes were small and confounding was pervasive (23–25). The few randomized trials addressing red meat intake found no significant effects on major cardiometabolic or cancer endpoints (24).

## The epigenetic role of beef intake

It has been reported that several bioactive compounds found in beef, particularly in grass-fed beef, influence epigenetic regulation through distinct mechanisms. The evidence is strongest for one-carbon metabolism nutrients, emerging for conjugated linoleic acid (CLA), and increasingly recognized for phytochemicals transferred from pasture to beef.

The most direct link between beef bioactive compounds and epigenetics runs through one-carbon metabolism, the biochemical network that supplies methyl groups for DNA and histone methylation. Beef is among the richest dietary sources of vitamin B12, a critical cofactor in this pathway along with folate, choline, betaine, and methionine (26, 27). Deficiencies in these methyl donors lead to aberrant DNA methylation patterns with consequences on gene expression. Maternal vitamin B12 deficiency in rat models altered DNA methylation of metabolically important genes in offspring, affecting metabolic programming. A human study found a positive correlation between circulating vitamin B12 levels and methylation of the mitochondrial D-loop region, the first evidence linking B12 to mitochondrial DNA methylation (28). The implications are both developmental and chronic. On the other hand, deficiencies in one-carbon metabolites during the periconception period lead to epigenetic alterations in DNA methylation and histone methylation in genes that regulate key developmental processes (28). Since beef provides B12, choline, and methionine in highly bioavailable forms, it contributes to the methyl donor pool that maintains normal epigenetic patterns.

Likewise, the conjugated linoleic acid (CLA), found predominantly in ruminant-derived foods like beef and dairy, modulates gene expression through mechanisms that intersect with epigenetic regulation. CLA activates PPARγ, a nuclear receptor that controls transcription of genes involved in inflammation, adipogenesis, and immune function. In a mouse model of inflammation-induced colorectal cancer, dietary CLA (1 g/100 g diet) prevented adenocarcinoma formation through a PPARγ-dependent mechanism while increasing regulatory T cells and suppressing TNF-*α* expression (30). Likewise, CLA’s anticancer mechanisms are primarily mediated by PPARγ activation and ferroptosis driven by lipid peroxidation (31). The t10,c12-CLA isomer inhibits the proliferation and migration of cancer cells by upregulating miR184 and miR215, microRNAs that contribute to cell cycle arrest, a form of epigenetic gene silencing mediated by non-coding RNA (32). The CLA also reduces cell proliferation and induces apoptosis in mammary, bladder, and colon cancer cell lines, with mechanisms involving modulation of the IGF-1R signaling pathway and lipoxygenase metabolites (33, 34). Grass-fed beef contains substantially higher CLA levels than grain-fed beef (35, 36). On the other hand, vaccenic (trans-11 C18:1) and rumenic (cis-9 trans-11 C18:2) acids reduce insulin resistance (37, 38). It has been suggested that vaccenic acid attenuates dyslipidemia, fatty liver, and inflammation (39) by reducing pro-inflammatory cytokines and platelet aggregation (40). Others researchers indicate that vaccenic acid has translational potential for the treatment of tumors and plays a critical role in children’s health (41, 42).

In addition, grass-fed beef contains phytonutrients transferred from pasture plants to animal tissues. Many researchers have demonstrated that when livestock eat diverse plants, terpenoids, phenols, carotenoids, and antioxidants become concentrated in meat, in quantities comparable to those in plant foods known to have anti-inflammatory, anti-carcinogenic, and cardioprotective effects (43). Similar studies comparing beef from Chilean pastures vs. Chilean feedlots have shown that these phytonutrients are even present in feedlot beef, because these systems include a higher proportion of roughage (~50%) in finishing diets than those reported for feedlots in the USA or Australia (usually <15%) (44). In addition, many of these phytonutrients are known to modulate epigenetic marks in other dietary contexts, including DNA methylation and histone acetylation, though direct studies on the epigenetic effects of phytochemicals specifically from beef consumption in humans are still lacking.

Quantitatively, grass-fed beef from biodiverse pastures contained higher levels of phenolic antioxidants (2.6-fold), alpha-tocopherol/vitamin E (3.1-fold), omega-3 fatty acids (4.1-fold), and phytochemical antioxidants (3.1-fold) compared to grain-finished beef (36–45). Another study found that grass-fed beef contained 3.1-fold higher levels of phytochemical antioxidants, with 2.9-fold more vitamin A and 4.2-fold more vitamin E, while grain-fed beef showed elevated homocysteine, a marker associated with disrupted one-carbon metabolism and aberrant methylation (46). Beef from steers finished in regenerative production systems in Chile recorded about 10-fold more flavonoid glycosides, 4-fold more carboxylic acids, and 3-fold more flavonoids than finished in a Chilean feedlot. In addition, they had 2-fold higher contents of hydroxybenzoic acids, hydroxycinnamic acids, phenolic alcohols, and flavanols (44).

While butirate, a short fatty acid, is primarily produced by gut microbial fermentation of fiber rather than consumed directly in beef, it represents a potent epigenetic modifier relevant to the broader diet-health picture. Butyrate is a well-characterized histone deacetylase (HDAC) inhibitor that drives epigenetic reprogramming in colon cancer cells by inhibiting DNMT1 (DNA methyltransferase 1), modulating CpG methylation, activating the mitochondrial TCA cycle, and upregulating tumor suppressor genes such as ABCA1 (47). In simple terms, it acts as a key postbiotic messenger with wide-ranging benefits for human health, especially in the gut but also beyond it. For example, it supplies about 70% of the energy needed by the cells lining your large intestine (colonocytes). This keeps the gut lining healthy and functioning well. It helps maintain a strong intestinal wall (“tight junctions”), reducing “leaky gut” and preventing bacteria or toxins from entering the bloodstream. It has anti-inflammatory effects, calming immune responses in the gut and potentially throughout the body. This may help with conditions like IBS, IBD (e.g., Crohn’s or colitis), and broader chronic inflammation. In addition, emerging research links it to better blood sugar control, metabolic health, immune balance, and even brain/mood benefits via the gut-brain axis (48, 49). In a diet-induced obesity model, butyrate enhanced histone acetylation at the promoters of circadian clock genes in muscle, upregulated fatty acid oxidation genes, and reduced weight gain (50).

Most evidence linking specific beef nutrients to epigenetic effects in humans is indirect, derived from cell culture, animal models, or observational associations. No randomized controlled trials have tested whether beef consumption *per se* alters human epigenetic due to controlled dietary studies are both expensive and difficult to run. Likewise, the phytochemical content of beef varies enormously across production systems, and the extent to which phytochemicals in beef reach biologically active concentrations in human tissues remains to be established. The most robust link remains the one-carbon metabolism pathway, in which beef’s contributions of B12, choline, and methionine to the methyl donor pool are well-characterized biochemically, even though the downstream epigenetic consequences of consuming beef specifically (versus other B12 sources) have not been isolated in human trials.

## Dichotomy of the production system (grass vs. grain-fed beef)

The grass-fed (pastoral) vs. grain-fed (industrial) framing of beef production, while useful shorthand, obscures a more complex reality. In many cases, especially in South American countries, beef production systems can be a mix between both; each configuration involves genuine trade-offs, and the optimal approach depends on what you are optimizing for. Globally, even in “intensive” beef production systems, forage accounts for more than 80% of an animal’s lifetime feed intake, with animals typically receiving high-grain diets only during a 3–4-month finishing period (51). Most beef cattle spend most of their lives on pasture before entering a feedlot phase, especially in industrial production systems. A comprehensive review done by Greenwood (52) underscores that extensive cow-calf and stocker systems remain the backbone of beef production worldwide, with feedlot finishing layered on top. This means the real comparison is rarely “pure pasture vs. pure feedlot” but rather different finishing strategies applied after a common pastoral foundation. An Irish study (Limousin × Charolais) comparing three systems: (a) grain-finished at 21 months, (b) silage-plus-grain at 24 months, and (c) forage-only at 28 months, found the last one offered improved profitability, superior fatty acid profiles, and used only inedible human feed, but required older slaughter ages and more land per unit of beef (53).

As mentioned previously, grass-finished beef consistently shows advantages in specific bioactive compounds. A 2024 metabolomics study found 907 of 1,575 measured compounds differed between pasture- and grain-finished beef (36). A 2025 study traced this advantage to a soil–plant–animal continuum: pasturelands had 1.4-fold higher organic matter, and grass-fed beef contained 3.1-fold more phytochemical antioxidants (46). On the other hand, grain-finished beef is not simply worse. It contained 14.6-fold higher levels of gamma-tocopherol and 1.3–1.5-fold higher levels of several B vitamins, including B5 and B6 (36). The mixed approaches, grass-fed with a short 45-day grain finish, can capture some advantages of both systems. This is a common practice among cattlemen in South American countries, such as Chile, where cattle are supplemented with cereal grains or an equivalent (e.g., molasses) in the pasture during the finishing phase (54), thereby improving fat color, fat covering, animal performance, and beef quality. Nevertheless, within grass-fed systems, variation is enormous. Pasture diversity matters as much as the grass-fed label itself: cattle grazing biodiverse, multi-species pastures concentrate a wider variety and greater amounts of phytochemicals in meat than cattle grazing monoculture pastures (55). Labeling confusion between “grass-fed” and “grass-finished” compounds the problem (55).

The environmental comparison between the two production systems is similarly complex. Production efficiency is higher, and greenhouse gas intensity per kg of beef is lower in intensive than extensive systems (51). Conventional life cycle analyses have consistently found higher greenhouse gases emissions per kg in grass-finished systems due to longer finishing times and enteric methane emissions. However, these analyses typically assume steady-state soil carbon stocks, ignoring potential carbon sequestration in well-managed grazing systems. Adaptive multi-paddock (AMP) grazing in Ontario showed sequestration rates of 0.957 Mg C/ha/yr., reducing the carbon footprint of beef by 65% when included in the accounting (56). Another study found that AMP grazing sequestered 3.59 Mg C/ha/yr., flipping the system’s carbon balance from net positive to net negative emissions (57). A 2025 systematic review, however, challenges these claims: across 28 studies comparing regenerative to conventional grazing, higher-strength cross-sectional and longitudinal studies showed median additional soil organic carbon (SOC) sequestration rates that were not significantly different from zero. The highest values were observed in lower-strength observational studies (58). This is an important caution. The carbon sequestration claims for regenerative grazing remain contested, and the strongest study designs do not yet support them. A Gulf Coast analysis captured the core dilemma: the least complex grazing system yielded the highest profit, while the most complex produced the lowest greenhouse gas impact, revealing a direct trade-off between economic and environmental goals (59).

The most promising path likely lies in integrated systems that combine the ecological benefits of pastoral management with targeted intensification. Soussana et al. (60) outline five principles for agroecological livestock production: improving animal health management, decreasing input dependency, reducing emissions, enhancing diversity for resilience, and preserving biodiversity. These principles reject both the feedlot monoculture and the romantic pastoral ideal in favor of systems designed around nutrient cycling and ecosystem function. Extensive pastures, when well-managed, can have higher net primary productivity, better soil N cycling, and greater C sequestration than unmanaged grasslands, while efficiently recycling nutrients through biotic processes. But above a threshold animal density, sustainable intensification requires crop-livestock integration (61, 62). Crop-livestock integration, using grains and crop residues as feed while returning manure nutrients to arable land, can raise the overall efficiency of both systems. A 2025 review by Moritz et al. (63) makes the conceptual case explicit: the traditional intellectual division between “ranching” in North America and “pastoralism” in Africa/Asia has limited understanding because of *a priori* assumptions. Livestock keepers worldwide “adopt, adapt to, and challenge” both capitalist logics and pastoral traditions, creating a diversity of hybrid systems that defy simple categorization (63).

## Future directions and research priorities

In January 2026, the U. S. A. 2025–2030 Dietary Guidelines placed meat, full-fat dairy, and vegetables at the broad top of a new inverted pyramid, nearly doubling protein recommendations to 1.2–1.6 g/kg body weight and explicitly naming red meat as a healthy protein source. This represents the most dramatic shift in federal nutrition policy in the U. S. A. in decades, and it simultaneously clarifies and complicates the research landscape for beef. The new 2025–2030 guidelines prioritize whole foods, limit ultra-processed foods and added sugars, and encourage cooking with butter or beef tallow. This represents a sharp departure from almost five decades of advice to limit saturated fat and red meat (64). The National Cattlemen’s Beef Association hailed the update, noting that red meat is specifically mentioned as a healthy source of protein in a varied, balanced diet. However, the shift is controversial (67, 68). Nutrition experts at Boston University cautioned that the guidelines “raise as many questions as they answer,” particularly regarding how the scientific evidence was interpreted. Some worry that encouraging more fats may conflict with existing evidence on saturated fat and cardiovascular risk (67, 68). This tension between the new U. S. guidelines and the EAT-Lancet planetary health diet, which recommended just 14 g/day of red meat, defines the central fault line in beef research going forward (69).

The evolution of human beings and society is transforming the role of beef production systems, moving from a traditional focus on “commodity volume” into a sophisticated narrative of ecosystem services and biological intelligence. This shift suggests that a steak is no longer just protein but a biological record of the animal’s environment, welfare, and genetic expression. In modern research, the pasture is viewed as a complex laboratory, and cattle are no longer seen merely as methane emitters but as potential tools for carbon sequestration. By managing grazing (such as AMP or regenerative grazing), researchers are finding that cattle can stimulate soil health, turning grasslands into massive carbon sinks that help mitigate climate change. This creates a circular narrative in which the animal’s presence restores the land it grazes, emphasizing the soil–plant–animal relationship (the terroir concept).

As a result of this terroir, epigenetics has emerged as a relevant area of development, as it acts as the “translator” of it. It is the biological memory of the production system. Science now shows that the management of an animal (what it eats, the stress it endures, and the climate it endures) leaves molecular “tags” on its DNA. These tags do not change the genetic code, but they act like a dimmer switch, turning certain genes up or down. A calf born to a well-nourished, low-stress mother is not just “lucky”; it is epigenetically programmed for better immunity and higher meat quality before it even takes its first breath.

In animal welfare, the narrative has moved beyond the simple absence of pain toward the pursuit of “positive affective states” that may have epigenetic effects. Researchers are using precision livestock farming (PLF) to understand and meet the animals’ needs in real time. When an animal is well-adapted and stress-free, its biology operates at optimal efficiency. This is not just ethical; it is a prerequisite for human health. Because when an animal is raised in a biodiverse, low-stress grazing system, the nutritional profile of the beef changes. The narrative of “red meat is bad” is being challenged by research into omega-3 enrichment, CLA levels, and plant-derived antioxidants. By focusing on sustainability and animal well-being, the industry is producing a “functional food” that provides more than just calories; it delivers specific health-protective compounds for human consumers. A summary of some areas of interest and potential areas of research is presented in Table 1. Undoubtedly, many areas herein have cross-cutting effects, impacting animal health and physiology, human health, product quality, and the sustainability of production systems.

**Table 1:** Summary of suggested areas for future research and main considerations.

Areas	Considerations	Future research
Human health	The most urgent research need is settling the methodological debate over how to grade nutritional evidence. The NutriRECS controversy revealed a fundamental disagreement: when GRADE criteria designed for pharmaceutical randomized controlled trials (RCTs) are applied to dietary evidence, the associations between red meat and chronic disease are rated as low certainty (73).	- Long-term RCTs comparing diets with different beef intakes on hard clinical endpoints (not just biomarkers).
		- Separation of unprocessed from processed meat effects, and dose–response characterization at moderate intakes.
		- Assessment of beef within whole dietary patterns rather than in isolation, since foods interact within dietary matrices.
Nutritional and functional value of beef	The complexity of the beef matrix makes difficult for other alternatives to equalize it at the metabolome level. It has been shown that the metabolite profiles of grass-fed beef and a leading plant-based alternative differed by 90% (171 of 190 profiled metabolites), despite apparent similarity in macronutrients declared in the labels (43). Some nutrients and metabolites like DHA, niacinamide, glucosamine, hydroxyproline, anserine, and squalene have been found only in beef (43). In addition, plant-based meat alternatives had significantly lower nutrient density scores (NRF5.3), lower protein (17 vs. 20 g/100 g), and far higher sodium (660 vs. 60 mg/100 g), with minimal fortification of nutrients associated with animal proteins (B12, vitamin D, iodine, zinc, omega-3 DHA) (74).	- Impact of beef production systems on bioactive compounds concentration (taurine, creatine, carnosine, CLA, heme iron).
		- Assessment of bioactive compounds found in beef on epigenetics responses in humans
		- Alternative meat foods and its impact on human health and sustainability
Production system–nutrition and animal welfare	The emerging science connecting pasture management to meat nutrient density opens a third research frontier. As reviewed earlier, grass-fed beef from biodiverse pastures contains 2–10-fold higher levels of phytochemicals, omega-3 s, and antioxidants. In addition, these pastures are more resilient and cope better with climate change (75).	- Standardized labeling and definitions for production systems (“grass-fed” vs. “grass-finished” vs. “hybrid (other) systems”).
		- Randomized controlled human feeding trials comparing health outcomes from grass-fed vs. grain-fed beef consumption.
		- Utilization of artificial intelligence and sensors to ensure cattle are thriving, not just surviving
Environmental accounting	Environmental research must move past greenhouse gases -per-kg metrics toward multi-dimensional assessments. The feed-food competition framework offers a more nuanced lens: studies considering systemic consequences propose larger reductions of chicken and pork (which consume more human-edible feed) while allowing a role for ruminants that convert non-edible biomass on non-arable land (76). Ruminant production plays a crucial role in the circular bioeconomy by upcycling agricultural by-products into nutritious food, approximately 85% of U. S. grazing land is unsuitable for crop production (77).	- Genetics and breeding (including genomic and epigenomic selection for methane-efficient animals),
		- Animal nutrition (methane inhibitors and feed additives),
		- Microbiome engineering to improve feed efficiency and reduce emissions.
		- Translational gut microbiome research is particularly promising, linking rumen microbial profiles to both meat quality and methane output

## Conclusion

Red meat, and beef in particular, has played a central role in shaping human evolution, not only through its contribution to energy and nutrient supply but also through its influence on metabolic regulation, potential epigenetic programming, and adaptive capacity. The integration of metabolomic and epigenetic perspectives reveals that beef should not be viewed as a static dietary component, but rather as a dynamic interface between ecosystems and human biology. Differences in production systems, particularly those influencing animal diets and environmental interactions, can modulate health outcomes in ways not captured by traditional nutritional metrics. The concept of nutritional-terroir livestock systems offers a novel framework for understanding these relationships (soil-plan-animal), emphasizing the need to consider food quality as an emergent property of ecological processes. Future research should prioritize integrative approaches that link agricultural practices to human health through mechanistic pathways, moving beyond reductionist models toward a systems-based understanding of nutrition.
